# Positive-case follow up for lymphatic filariasis after a transmission assessment survey in Haiti

**DOI:** 10.1371/journal.pntd.0010231

**Published:** 2022-02-25

**Authors:** Marisa A. Hast, Alain Javel, Eurica Denis, Kira Barbre, Jonas Rigodon, Keri Robinson, Tara A. Brant, Ryan Wiegand, Katherine Gass, Marc Aurèle Telfort, Christine Dubray

**Affiliations:** 1 Centers for Disease Control and Prevention, Atlanta, Georgia, United States of America; 2 IMA World Heath, Port-au-Prince, Haiti; 3 Neglected Tropical Diseases Support Center (NTD-SC), Task Force for Global Health, Atlanta, Georgia, United States of America; 4 Centers for Disease Control and Prevention Country Office, Port-au-Prince, Haiti; 5 Ministry of Public Health and Population (MSPP), Port-au-Prince, Haiti; Swiss Tropical and Public Health Institute, SWITZERLAND

## Abstract

**Background:**

Lymphatic filariasis (LF) has been targeted for global elimination as a public health problem since 1997. The primary strategy to interrupt transmission is annual mass drug administration (MDA) for ≥5 years. The transmission assessment survey (TAS) was developed as a decision-making tool to measure LF antigenemia in children to determine when MDA in a region can be stopped. The objective of this study was to investigate potential sampling strategies for follow-up of LF-positive children identified in TAS to detect evidence of ongoing transmission.

**Methodology/Principle findings:**

Nippes Department in Haiti passed TAS 1 with 2 positive cases and stopped MDA in 2015; however, 8 positive children were found during TAS 2 in 2017, which prompted a more thorough assessment of ongoing transmission. Purposive sampling was used to select the closest 50 households to each index case household, and systematic random sampling was used to select 20 households from each index case census enumeration area. All consenting household members aged ≥2 years were surveyed and tested for circulating filarial antigen (CFA) using the rapid filarial test strip and for Wb123-specific antibodies using the Filaria Detect IgG4 ELISA. Among 1,927 participants, 1.5% were CFA-positive and 4.5% were seropositive. CFA-positive individuals were identified for 6 of 8 index cases. Positivity ranged from 0.4–2.4%, with highest positivity in the urban commune Miragoane. Purposive sampling found the highest number of CFA-positives (17 vs. 9), and random sampling found a higher percent positive (2.4% vs. 1.4%).

**Conclusions/Significance:**

Overall, both purposive and random sampling methods were reasonable and achievable methods of TAS follow-up in resource-limited settings. Both methods identified additional CFA-positives in close geographic proximity to LF-positive children found by TAS, and both identified strong signs of ongoing transmission in the large urban commune of Miragoane. These findings will help inform standardized guidelines for post-TAS surveillance.

## Author summary

Lymphatic filariasis (LF) is a debilitating parasitic disease that has been targeted for global elimination. The transmission assessment survey (TAS) is a tool used to determine if LF transmission has reached low enough levels that prevention activities can be stopped. This study aimed to identify methods to investigate positive LF cases found during TAS. The investigation was conducted in Nippes Department, Haiti, where 8 positive cases were found in TAS in 2017. Participants were recruited through two methods: purposive selection of the closest 50 households to the positive case, and random selection of 20 households in the census enumeration area of the case. Participants completed a survey and were tested for LF antigen, indicative of current infection, and parasite-specific antibody, indicative of current or past infection. A total of 1,927 people participated in the study; 1.5% of these were antigen-positive, and 4.5% were antibody-positive. Purposive sampling found a higher number of antigen-positive individuals, and random sampling found a higher percent positive. Both sampling methods were feasible to use in this setting, and both methods identified signs of ongoing transmission in a large urban area. Additional research is needed to help standardize guidance for post-TAS surveillance to best identify ongoing transmission.

## Introduction

Lymphatic filariasis (LF) is a mosquito-borne parasitic disease caused by the filarial worms *Wuchereria bancrofti*, *Brugia Malayi*, *and B*. *timori*, and is endemic to tropical areas in 72 countries [1]. The debilitating clinical disease caused by LF can result in fluid accumulation in the extremities, resulting in lymphedema, hydrocoele, and acute adenolymphangitis. LF is one of the leading causes of chronic disability worldwide, and it is estimated that LF was responsible for over 5 million disability-adjusted life years prior to the implementation of disease control programs [2]. Due to this high global burden, LF has been targeted for global elimination as a public health problem since 1997 [3].

The primary strategy for LF control and elimination is the use of annual mass drug administration (MDA) for the entire population at risk for at least five consecutive years or until local transmission is interrupted [4]. In order to define when this criteria has been reached and MDA can be stopped in a region, the transmission assessment survey (TAS) was developed as a decision-making tool to determine when prevalence of LF has reached low enough levels that transmission cannot be sustained, even in the absence of active control measures [5].

The current World Health Organization (WHO) criteria states that TAS can be initiated once pre-TAS sentinel site and spot checks show <2% LF antigenemia among the population over 5 years old in a given evaluation unit (EU), which is typically a district or a combination of districts [4]. As part of TAS, antigenemia in children aged 6–7 is systematically measured, typically using a cluster survey of at least 30 schools, although some variations exist globally. Measuring infection in children this age is relevant for LF since they are of sufficient age to develop a mature parasite but young enough for a current infection to indicate recent transmission. WHO guidelines then state that MDA can be stopped in the EU if prevalence of LF antigenemia among children this age in the first TAS (TAS 1) is below 2% in regions where *Anopheles* or *Culex* mosquitoes are the main vectors or below 1% where *Aedes* mosquito species are the main vectors [4]. Once antigenemia falls below the appropriate threshold and MDA is stopped, the EU must then pass 2 additional TAS (TAS 2 and TAS 3) over a period of 4–6 years. Once all EUs in a country successfully pass three TAS, the country is eligible to submit a dossier to the WHO to receive formal acknowledgement of validation of the elimination of LF as a public health problem, indicating that prevalence in the population is below the threshold to support onward transmission [6].

Since its inception in 2011, TAS has been widely integrated into national elimination programs worldwide, with over 1,000 EUs having already passed TAS 1 [7]. However, a continuing challenge for LF programs is how to interpret and respond to antigen-positive children identified in a TAS 2 or 3 survey that passes the overall threshold. Although a passing result suggests that transmission has been interrupted, any positive result in young children without travel to other regions is cause for concern regarding ongoing transmission, particularly if those cases are clustered geographically or if the number of cases increases between surveys [8]. Furthermore, there is some concern that a cluster-based survey might not be sensitive enough to detect all hotspots of transmission, particularly in large or environmentally heterogeneous EUs [9,10].

Due to these concerns, the WHO currently encourages program managers to conduct follow-up surveys in communities where antigen-positive children are detected [4]. However, little guidance exists on how follow-up should be conducted, what threshold should trigger a programmatic response, or what this response should entail. Given the recent evidence of residual LF transmission or resurgence in Sri Lanka and American Samoa following multiple passed TAS, additional research is needed to help guide these programmatic decisions and better determine the utility of TAS for measuring interruption of transmission [11–13]. Additional research is also needed to determine the role of emerging tools, including the use of antibody testing which may detect both current and past infection, and how these complement antigen detection in the context of identifying ongoing transmission.

Haiti is one of only four countries in the Americas where transmission of *W*. *bancrofti* still occurs [7]. In 2001, it was determined that LF endemicity was widespread throughout the country, and the decision was made to conduct MDA nationwide. By 2012, MDA was conducted in all 140 communes (equivalent to districts) [14]. As of 2020, 122 of these communes had reached the WHO criteria to stop MDA, many of which had low or moderate LF transmission at baseline. However, the remaining 18 communes have had persistent LF transmission despite more than 10 years of MDA, and other communes have had an increase in antigen-positive children despite continuing to pass their TAS. In the context of potential continuing transmission, it is critical to follow-up positive cases from TAS 2 and TAS 3 to identify any areas where targeted MDA may be warranted to sustain the gains made towards LF elimination. The objective of this study was to identify potential sampling strategies for positive case follow-up after a TAS using antigen and antibody detection that optimizes the chances of correctly identifying evidence of ongoing transmission in Haiti, while saving program resources.

## Methods

### Ethics statement

This study was approved by the National Bioethical Committee in Haiti (1718–84) and the Scientific Internal Review Board at the CDC (2018–430). Formal informed consent was obtained by all participants or parents/guardians of children <18 years old. All study participants were read a consent form in Haitian Kreyol by the survey team and provided verbal informed consent or parental consent, and children aged 7–17 years provided verbal informed assent. A written copy of the consent form in Haitian Kreyol and the study investigator’s phone number was left with each household. Consent for collecting geographic coordinates was obtained from the head of household. All data were stored on secure servers and password-protected computers. Results of biological testing were kept confidential and only were shared with the study participant or their guardian. Individuals with positive test results were offered treatment with the standard of care in Haiti.

### Study site

The site for this study was the department of Nippes, Haiti, which has a population of approximately 342,000 and is comprised of 11 communes [15]. The most populous commune is Miragoane, which has more than 62,000 residents and contains the department capital. Due to a low baseline prevalence of <5%, all communes were combined into one EU for the LF elimination program, so the whole department is evaluated for TAS together. The department successfully received five consecutive rounds of MDA from 2009–2013 with sufficient coverage, defined as ≥65% of the population. TAS 1 was conducted in 2015 using a cluster survey of 30 primary schools, and two children were identified as antigen-positive using the rapid filariasis test strip (FTS) (Alere, Scarborough, ME), which detects circulating filarial antigenemia (CFA) for adult worms. This was below the critical cutoff of 2% for regions with *Culex* vectors, and MDA in the department was stopped. Nippes underwent TAS 2 in 2017 and passed again, however the number of CFA-positive children had increased to eight. Of these, four came from the commune of Miragoane, with two residing across the street from each other in a dense urban area. The four other CFA-positive children were located in the communes of Anse-a-Veau, L’Asile, Petit-Trou de Nippes, and Plaisance du Sud.

### Study design and sampling

**Fig 1:**
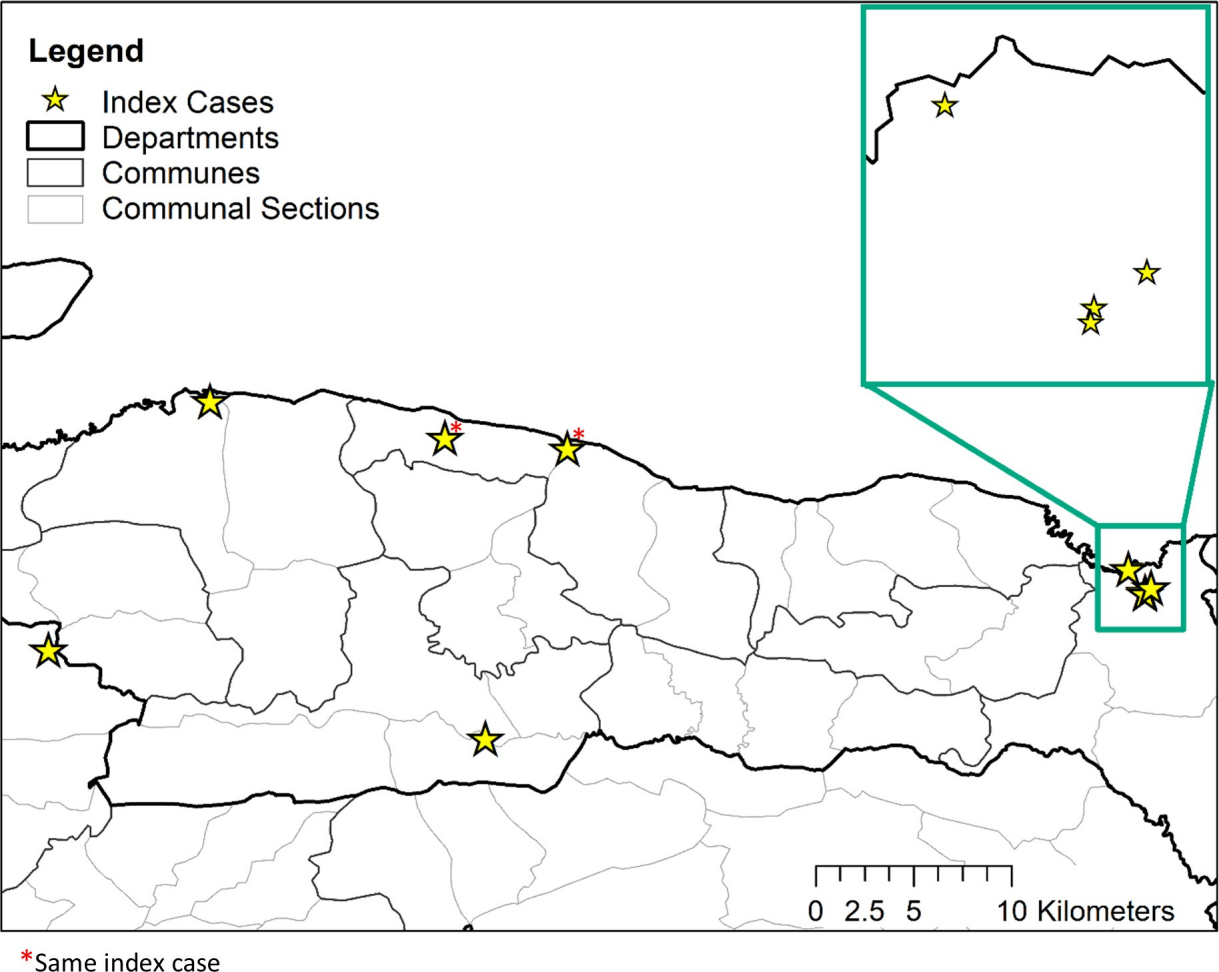
Locations of antigen-positive lymphatic filariasis cases identified in TAS 2, Nippes Department, Haiti 2017. Exact locations are jittered for participant confidentiality. Shapefiles for administrative boundaries are from the Centre National d’Information Geo-Spatiale and are available at https://data.humdata.org/dataset/hti-polbndl-adm1-cnigs-zip.

Each CFA-positive child identified in TAS 2 was considered an index case. The residential locations of the index cases were plotted in ArcGIS Version 10.7 (ESRI, Redland, CA), and each case was mapped to an Enumeration Area (EA) for follow-up (Fig 1). EA boundaries were previously determined by the Haitian Ministry of Health and Ministry of Statistics for use with the census and the Demographic Health Survey and range from 0.02–30.8 square kilometers. The index case in Anse-a-Veau had moved recently from a more rural area, so both the case’s current location and previous residence were mapped to their respective EAs for follow-up. Purposive and random sampling methods were used to select participants for this study.

### Purposive sampling

For each index case, the location of the index case household was identified by the field team using global position system (GPS) coordinates. The index household and the 50 closest households by straight-line distance were selected for inclusion in the sample. Fifty was chosen since it was the maximum deemed to be feasible and sustainable using this method. In all selected households, head of household consent was obtained, and household coordinates were collected on electronic mobile devices. If any of the 50 nearest households declined, it was replaced with the next nearest household until 50 had been enrolled.

To identify the 50 nearest households to each index case, structures surrounding index case households were enumerated using high-definition satellite images (DigitalGlobe, Denver, CO), and the proportion of inhabited structures in that EA was calculated using geo-linked census data. Using a projected non-response/refusal rate of 10%, an approximate radius was calculated around each index household to encompass the 50 households anticipated to participate. A buffer of the appropriate radius was developed in ArcGIS for each index case, and these shapefiles were loaded onto Garmin GPSMAP 64 handheld GPS devices (Olathe, KS) and Locus Map software (Prague, Czech Republic) for use by the field team. Once the index household was identified, field teams started at the index house and proceeded outwards in concentric circles until 50 households had been enrolled, using the buffer shapefile as a guide. Due to their close proximity, the two Miragoane index cases in the same EA were treated as a unit and a purposive circle was drawn around the midpoint between the two houses.

### Random sampling: Index EA

In each index case EA, field teams conducted a comprehensive census in the EA to identify and number all occupied households. The boundaries of the EA were determined using maps and GPS tools, and every structure in the EA was categorized as a household or non-household. Each occupied household was given a number, and 20 households were selected for inclusion using systematic random sampling, which was the maximum deemed to be feasible and sustainable using this method. A sampling interval was determined by dividing the total number of households by 20 and rounding down to the nearest whole number. A random start was selected between 1 and the sampling interval using random number generating software. A list of selected households was developed by serially adding the sampling interval to the random start until 20 households had been selected. At each selected house, head of household consent was obtained, and household coordinates were collected on electronic mobile devices. If any of the selected households declined to participate, it was not replaced.

### Random sampling: Neighbor EA

If any participant in a selected household from either purposive or random sampling tested CFA-positive for LF (methods to follow), additional random sampling of 20 households was conducted in each of the two neighboring EAs nearest to the positive case. For these neighboring EAs, sampling methods were identical to random sampling conducted in index case EAs, including conducting the census, household numbering, identification of a sampling interval and random start, listing of selected households, and head of household consent.

### Field methods and sample collection

Field data collection occurred from July to August 2019. In selected households, all household members aged 2 years and older were invited to participate in the sample. Adults provided informed consent or parental permission for their children under 18 years, and children aged 7–17 years provided informed assent. Exclusion criteria included age under 2 years, having another primary residence, and inability to provide informed consent due to physical or mental incapacity. All consenting household members were given a unique barcode and administered an electronic questionnaire using Secure Data Kit software (Atlanta, GA). The questionnaire collected information on participant demographics, time living in the EA, history of travel, participation in the most recent MDA, and use of bed nets.

All consenting participants provided approximately 250μl of blood, which was collected by finger stick into heparinized collection tubes. Collection tubes were marked with a matching barcode to the participant and were not otherwise marked with any personally identifying information.

Antigen testing was done at point-of-collection with FTS using standardized methods [16]. In brief, 75 μL of blood were pipetted from the heparinized tubes onto the test sample pad and allowed to flow through the strip. After exactly 10 minutes, the result window was read for a positive, negative or invalid CFA result. Any positive or invalid FTS were repeated with a second confirmatory FTS. Individuals with two positive FTS were counted as CFA-positive, and individuals with one or more negative FTS were counted as CFA-negative. Tubes with the remainder of the blood samples were then transported to the field laboratory in a portable cooler, where dried blood spots (DBS) were prepared for each participant. Ten μL of heparinized blood were pipetted onto each of six extensions of Trop Bio filter paper (Cellabs, Sydney, Australia) for a total sample of 60 μL. Filter paper was labeled with the unique barcode for the participant, allowed to dry, and packed with desiccant in labelled bags until transport to the National Laboratory of Public Health in Port au Prince, where they were stored at -20°C.

Individuals with two positive FTS were treated with the standard combination of albendazole and diethylcarbamazine (DEC), with dosage calculated based on the age of the participant following standard procedure in Haiti. Pregnant women were eligible for participation in the study; however, due to the unknown effects of these medications in utero, pregnant participants who tested CFA-positive were advised to seek treatment for LF a week after delivery.

### Laboratory analysis

Preserved DBS were transported to the Centers for Disease Control and Prevention (CDC) in Atlanta, GA where they were tested for IgG4 antibodies against the recombinant Wb123 antigen using the Filaria Detect IgG4 ELISA kit (InBios, Seattle, WA), a direct enzyme immunoassay. The assay was performed according to the standard operating procedure provided by the manufacturer with minor modifications, as described previously [17]. In brief, blood spot extensions of DBS and positive and negative controls were diluted 1:50 in kit-provided sample buffer and stored overnight at 4°C. Samples and controls were added to plate wells, sealed, and incubated at 37°C for 30 minutes, washed using an automated plate washer and kit-provided wash buffer, incubated again with mouse anti-human IgG4 conjugated with horseradish peroxidase for another 30 minutes, and washed again. Plates were developed at room temperature in the dark for 13 minutes with an added 100 μL of tetramethylbenzidine substrate in each well, stopped using kit-provided stop solution, and incubated for one minute. Plates were read on a microplate reader at 450 nm. To compare optical density (OD) values across plates the OD values were normalized by dividing the mean OD of the sample by the mean OD of the H3-positive control from the same plate. Additional details are described in Supplementary Materials S1 Text.

### Data analysis

#### Cutoff determination for ELISA OD values

In order to analyze the ELISA OD values as a dichotomous variable, a fixed finite mixture model (FMM) [18] was fit to the serology OD dataset to determine a cutoff using the *flexmix*, *mixtools*, *mixsmsn* and *sn* packages in R version 4.0.2 (R Core Team, Vienna, Austria). These models use maximum likelihood estimation to fit a two-component FMM to the data to estimate the parameters of “seropositive” and “seronegative” distributions [19,20]. Each component was fitted with a normal or skew-normal distribution to log-transformed OD values and assumed to be independent of age. The ELISA value at which the probability of positive was greater than 0.5 was used as the cutoff, which is also the point where the two distributions intersect [21]. Values above the cutoff were determined to be seropositive and values below were considered seronegative.

#### Statistical analysis

All analyses were done in Stata 13.1 (Stata Corporation, College Station, TX) and R version 4.0.2 statistical software. Frequencies of demographic and behavioral characteristics of the study population were calculated. Both CFA and serology results are described by participant characteristics and sampling method, and bivariate comparisons were conducted using chi-squared and Fisher’s exact tests. For purposively sampled households, the proportion and number CFA positive were compared by increasing number of sampled households from the index case house (e.g., closest 10, closest 20, etc.). To better visualize their distribution, the natural log of serology OD values were plotted against participant characteristics in scatterplots.

Exploratory analyses were conducted both by index case and by geographic zone. Index cases were defined by number as cases 1–8. The two index cases in the same EA in Miragoane, designated as 1a and 1b, were analyzed together as index case 1 due to their close proximity and resulting inability to differentiate in sampling. Geographic zones were defined as Miragoane (containing index cases 1a, 1b, 2, and 3), L’Asile (index case 4), Plaisance du Sud (index case 5), Petit-Trou de Nippes (index case 6), Anse-a-Veau rural (index case 7), and Anse-a-Veau urban (index case 8). As described above, index cases 7 and 8 were the same individual but were analyzed separately since he or she resided in the two zones at different times.

## Results

### Study population

A total of 1,927 participants from 786 unique households completed the survey and FTS testing. Of these, nearly 40% were male and 18% were under age 10 years (Table 1). Nearly half of participants resided in the commune of Miragoane, with 8–14% residing in each of the other 5 geographic zones. Forty percent of participants were students, 20% listed commerce as their primary occupation, and 15% did not have a stated occupation. Approximately one fifth of participants reported sleeping under a bed net the previous night, and a quarter reported travel outside their commune in the past year. Among participants aged 12 and above who would have been eligible to take MDA during the last administration, 65% reported ever taking medications for LF as part of MDA in the past.

**Table 1:** Demographics and participant characteristics among survey respondents in Nippes Department, Haiti, July-August 2019. N = 1,927.

	n	%
**Sex**		
Male	742	38.5%
Female	1,185	61.5%
**Age in years**		
<10	351	18.2%
10–19	461	23.9%
20–44	702	36.4%
≥45	413	21.5%
**Commune of residence**		
Miragoane	861	44.7%
L’Asile	268	13.9%
Plaisance du Sud	158	8.2%
Petit-Trou de Nippes	152	7.9%
Anse-a-Veau rural	268	13.9%
Anse-a-Veau urban	220	11.4%
**Occupation**		
Student	776	40.3%
Commerce	378	19.6%
Agriculture^1^	157	8.1%
Manual labor	71	3.7%
Public sector	32	1.7%
Private sector	19	1.0%
None	288	15.0%
Other	86	4.5%
Missing	120	6.2%
**Slept under bed net last night**		
Yes	392	20.3%
No	1,535	79.7%
**Traveled outside commune past year**		
Yes	500	26.0%
No	1,427	74.0%
**Have taken MDA in past** ^2^		
Yes	943	64.7%
No	488	33.5%
Don’t know	26	1.8%
**Sampling method** ^3^		
Purposive	1,221	63.4%
Random (index)	381	19.7%
Random (neighbor)	500	25.9%
**Total**	**1927**	**100%**

^1^ Includes farming or fishing

^2^among participants aged 12 years and above

^3^ adds to more than 100% because 175 participants were from households selected for both purposive and random sampling; MDA = mass drug administration

By mode of sampling, 63% of participants were selected by purposive sampling, 20% were selected by random sampling in the index case EA, and 26% were selected by random sampling in a neighboring EA. Nine percent of participants (175) were selected for both purposive and either index case EA or neighbor EA random sampling, and thus these numbers add to more than 100%.

### Antigen results (CFA)

**Fig 2:**
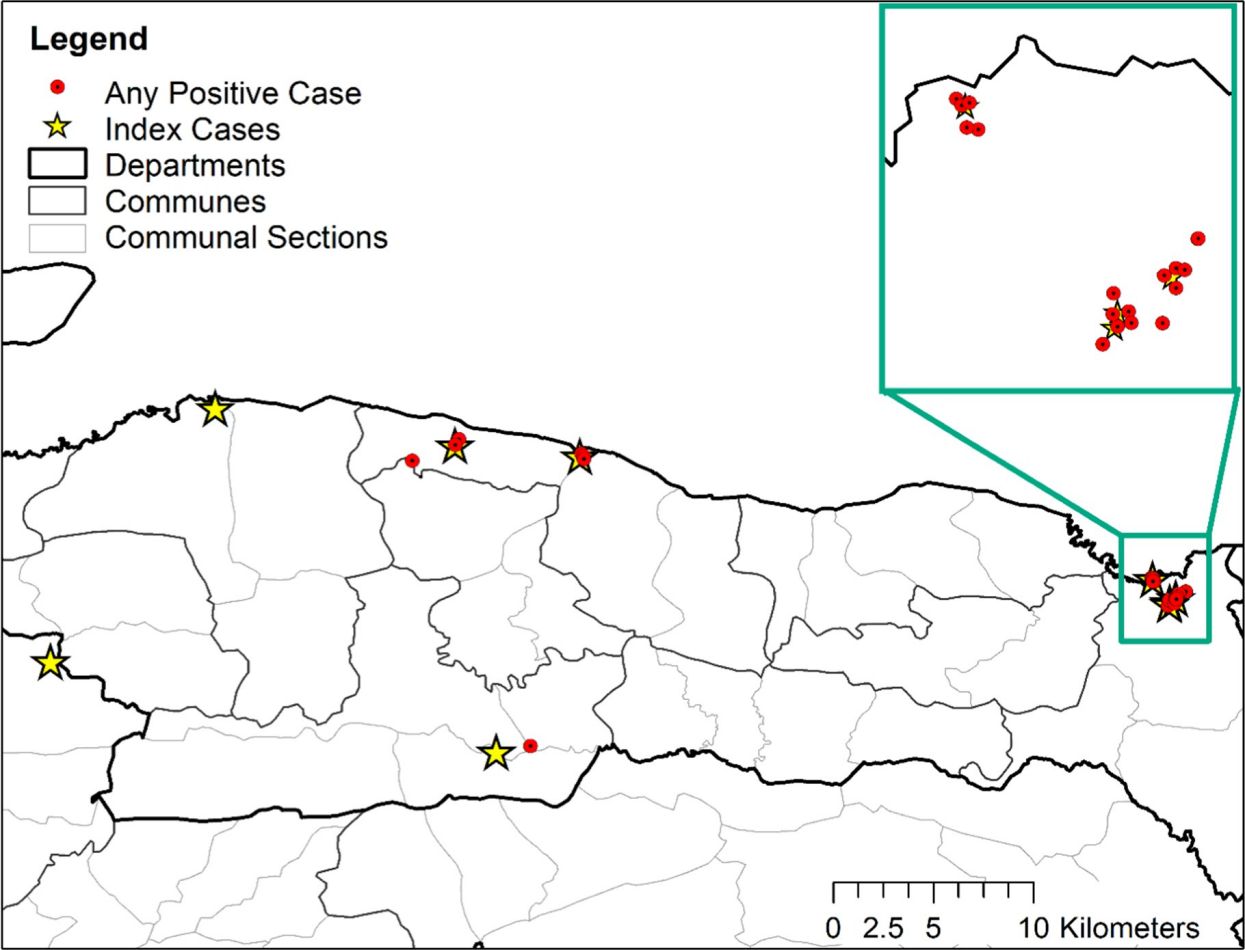
Locations of individuals positive for circulating filarial antigen (CFA) identified through purposive or random sampling in relation to their index case, Nippes Department, Haiti July-August 2019. Exact locations are jittered for participant confidentiality. Shapefiles for administrative boundaries are from the Centre National d’Information Geo-Spatiale and are available at https://data.humdata.org/dataset/hti-polbndl-adm1-cnigs-zip.

Of the 1,927 participants who completed FTS testing, 29 were CFA-positive, for a total positivity rate of 1.5% (Table 2). Of these, 21 (72%) were from Miragoane, and each index case in this commune led to the identification of CFA-positive participants, with the number found per index case ranging from 6 to 8 (Fig 2). An additional 3 and 4 CFA-positive participants were found in Anse-a-Veau rural and urban zones, respectively, 1 was found in L’Asile, and no CFA-positive participants were found in Plaisance du Sud or Petit-Trou de Nippes (S1 Table). Overall, 6 of the 8 original index cases (75%) were linked to at least one CFA-positive participant. Miragoane also had the highest rate of CFA positivity, with 2.4% of participants testing positive compared to the range of 0.0%–1.8% in other geographic zones (Chi squared *P* value = 0.03).

**Table 2:** Filariasis test strip (FTS) and serology results by participant characteristic, Nippes Department, Haiti, July-August 2019. FTS N = 1927; Serology N = 1,914.

	CFA Positive n (%)	P value*	Serology Positive n (%)	P value*
Sex				
Males	12 (1.6%)	0.7	43 (5.8%)	*0*.*02*
Females	17 (1.4%)		44 (3.7%)	
Age in years				
<10	2 (0.8%)	0.08	11 (3.1%)	0.06
10–19	5 (1.1%)		14 (3.0%)	
20–44	17 (2.4%)		35 (5.0%)	
≥45	5 (1.2%)		27 (6.6%)	
Slept under bed net last night				
Bed net	4 (1.0%)	0.4	9 (2.3%)	*0*.*02*
No bed net	25 (1.6%)		78 (5.1%)	
Travel outside commune past year				
Yes	8 (1.6%)	0.8	27 (5.4%)	0.2
No	21 (1.5%)		59 (4.2%)	
Have taken MDA in past				
Yes	14 (1.5%)	0.3	42 (4.5%)	0.9
No	11 (2.3%)		22 (4.6%)	
Geographic zone				
Miragoane	21 (2.4%)	*0*.*03*	43 (5.1%)	0.5
L’Asile	1 (0.4%)		9 (3.4%)	
Plaisance du Sud	0 (0.0%)		6 (3.8%)	
Petit-Trou de Nippes	0 (0.0%)		9 (6.0%)	
Anse-a-Veau rural	3 (1.1%)		13 (4.8%)	
Anse-a-Veau urban	4 (1.8%)		6 (2.7%)	
Sampling method				
Purposive sampling	17 (1.4%)	0.08	62 (5.1%)	0.2
Random (index)	9 (2.4%)		15 (4.0%)	
Random (neighbor)	3 (0.6%)		16 (3.2%)	
**Total**	**29 (1.5%)**		**87 (4.5%)**	

*P values were Chi squared tests or Fisher’s Exact tests as appropriate; CFA = circulating filarial antigen

Two CFA positives were found among participants aged <10 years (positivity 0.8%), both of which resided in Miragoane and were between the ages of 4 and 6 years. CFA positivity rates were slightly higher among participants aged 20–44 years (2.4%) compared to other age bands; however, this difference was not statistically significant (*P* = 0.08). Positivity was slightly higher among participants who did not sleep under a bed net (1.6% vs. 1.0%) or did not take MDA in past (2.3% vs. 1.5%) compared to those who did; however, these differences also were not statistically significant (*P* = 0.4 and 0.3, respectively). Rates of positivity were similar by participant sex and history of travel.

Purposive sampling identified 17 CFA-positive participants for a positivity rate of 1.4%, random sampling in the index case EA identified 9 positive participants for a positivity rate of 2.4%, and random sampling in a neighboring EA identified only 3 positive participants for a positivity rate of 0.6% (Table 2). Thereby, purposive sampling identified the highest number of CFA-positive participants, but random sampling in the index case EA returned the highest percent positive. Among the 6 index cases that yielded any CFA-positive cases in this investigation, purposive sampling found positives for 5 index cases in 3 geographic zones (Miragoane, rural and urban Anse-a-Veau), and random sampling in the index EA found positives for 4 index cases in 3 geographic zones (Miragoane, L’asile, urban Anse-a-Veau) (S1 Table). Both of these sampling methods missed cases in one zone each. Positivity rate by geographic zone ranged from 1.3–2.7% for purposive sampling and 2.0–9.6% for random sampling. Random sampling in a neighboring EA found one additional CFA-positive participant for 3 of the 6 index cases.

**Fig 3:**
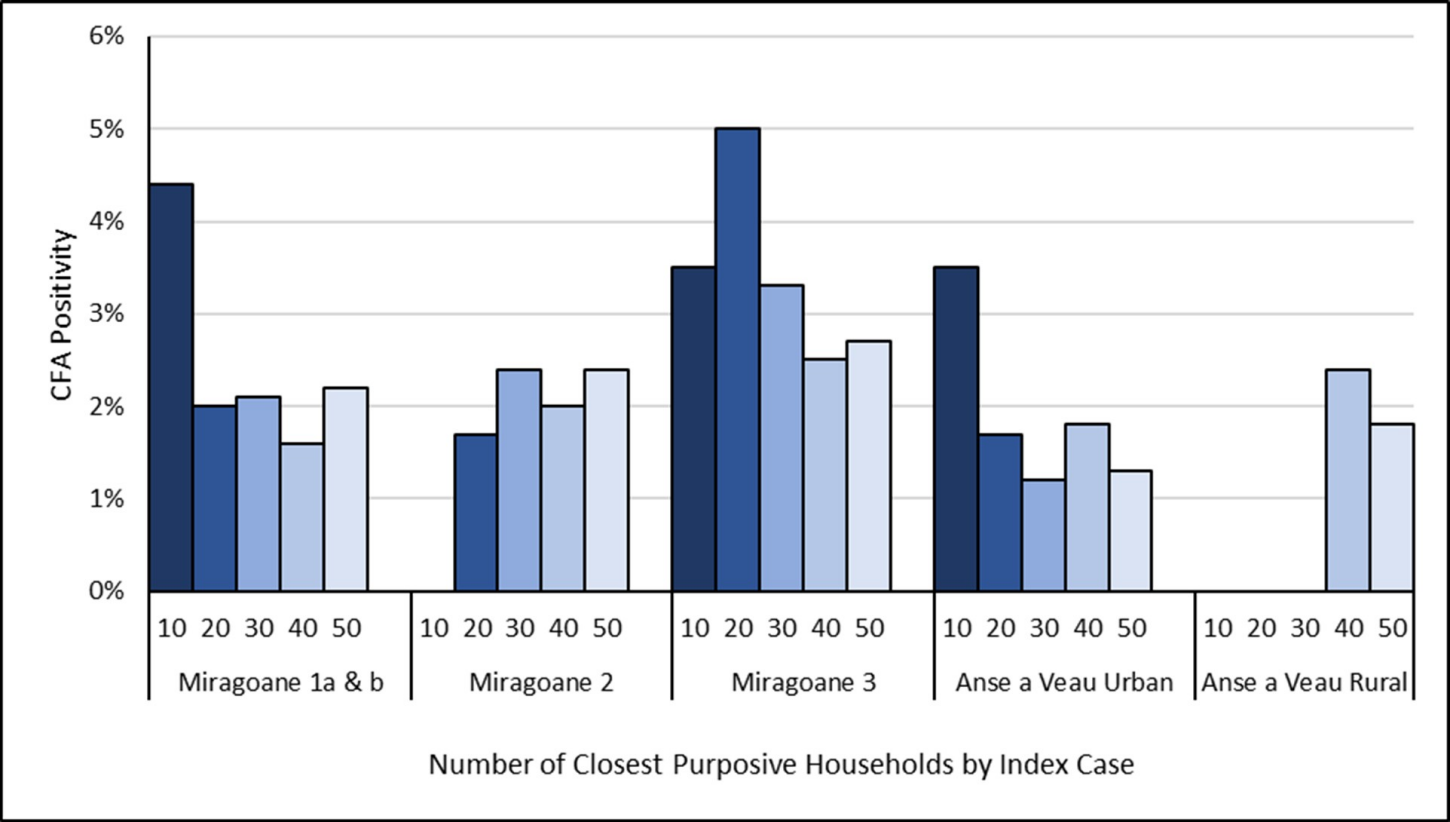
Circulating filarial antigen (CFA) prevalence by geographic zone, index case, and number of purposively sampled households by increasing distance from index case household, Nippes Department, Haiti, July-August 2019.

Among purposively sampled households, average CFA positivity was highest in the closest 10 households to the index case at 1.6%, declined to 1.4% in the 20 closest households, and then remained relatively stable at 1.3%, 1.3%, and 1.4% in the closest 30, 40, and 50 households respectively. However, these differences were not statistically significant, and there was substantial variation by index case and geographic zone (Figs 3 and S2). For 4 of 5 index cases with purposively identified CFA-positive participants, the majority of these were found within the 20 closest households to the index household, and prevalence in these households was higher than random sampling for 3 of 5 index cases (S1 Table and S1 Fig). The primary exception to this was for Miragoane index case 2, which had >9% positivity among randomly sampled participants compared to 2.4% positivity among purposively sampled households.

### Serology results (ELISA OD values)

Of the 1,927 participants who received FTS testing, 1,914 (99.3%) also provided DBS for serology analysis. Normalized mean OD values from ELISA testing ranged from 0.04 to 4.5, with a mean of 0.11. The cutoff for serology positivity determined by FMM methods was 0.18. Using this cutoff, there were 87 serology-positive participants, for a seropositivity rate of 4.5%. Concordance with CFA antigen results was 95%, with 6 participants positive by both methods, 1,813 negative by both methods, 23 CFA-positive only, and 81 seropositive only.

**Fig 4:**
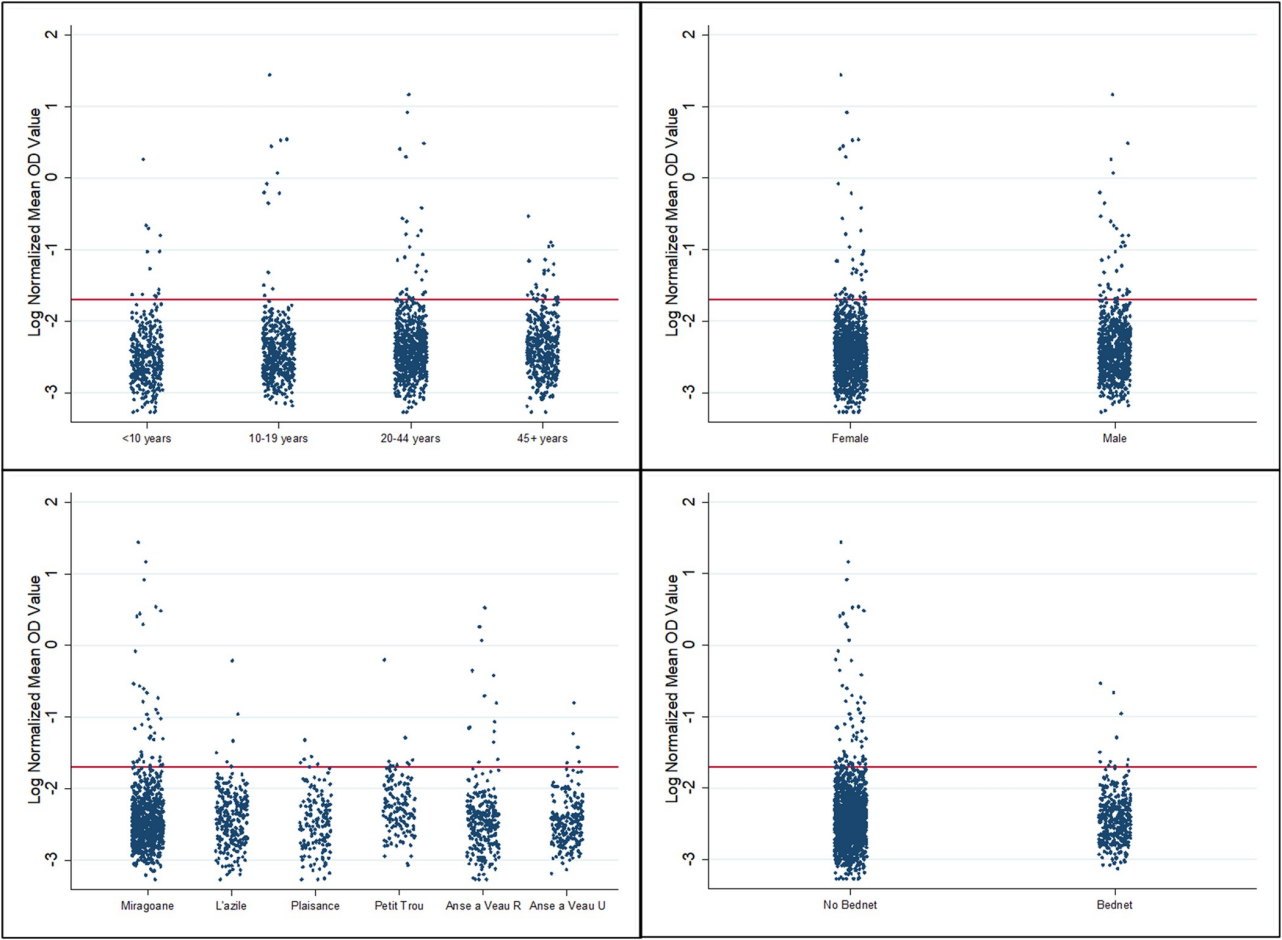
Log normalized mean optical density (OD) values for IgG4 antibodies against the recombinant Wb123 antigen by participant characteristics. Red line indicates cutoff for seropositivity.

Seropositivity was higher than CFA positivity across every category, however the size of the difference varied (Table 2). Seropositive participants were found for all 8 index cases, with positivity rates ranging from 2.7–6.0% (S2 Table). The distribution of log-transformed OD values across participant characteristics can be seen in Fig 4. The highest number of seropositive participants was found in Miragoane (43, 5.1% positive), but the highest percent seropositive was found in Petit-Trou de Nippes (6.0% positive), which had no CFA positives. There were no significant differences in seropositivity by index case or geographic zone (Table 2 and Fig 4).

Eleven seropositive results were found in participants <10 years (3.1%), and seropositivity increased by age to 6.6% in participants aged ≥45 years, although this increase was not statistically significant (Table 2). Seropositive children were aged 4–9 years and were located in Miragoane, rural Anse-a-Veau, and Petit-Trou de Nippes. Seropositivity was higher among male (5.8%) than female (3.7%) participants (*P* = 0.02) and was higher among participants who did not sleep under a bed net (5.1%) than those who did (2.3%) (*P* = 0.02) (Table 2 and Figs 4 and S3). Seropositivity was similar among participants who did or did not participate in MDA for LF in the past. By sampling method, seropositivity was 5.1% by purposive sampling, 4.0% by random sampling in the index case EA, and was 3.2% by random sampling in neighboring EAs. Purposive sampling found at least one seropositive participant for all 8 index cases, random sampling in the index EA found seropositive participants for 7 of 8, and random sampling in neighboring EAs found seropositive participants for 4 of 8 index cases.

## Discussion

This investigation describes findings for several methods of follow-up for CFA-positive children identified in TAS 2 in the Department of Nippes, Haiti. From 8 initial index cases in 6 geographic zones, a total of 29 additional CFA-positive individuals were identified out of 1,927 tested, and CFA-positives were found during follow-up for 6 of the 8 index cases. High rates of CFA positivity were found in the commune of Miragoane (2.4%).

Overall, both purposive and random sampling methods detected additional CFA-positive persons in the vicinity of most index cases, and both methods identified a high positivity rate in Miragoane. Purposive sampling tested, found, and treated the highest number of CFA-positives, and this method found follow-up CFA-positive participants for a higher number of index cases. Correspondingly, random sampling in the index case EA returned the highest percent positive and was therefore most efficient at identifying additional cases. Random sampling in neighboring EAs returned both a low number and percent positivity, indicating that this additional element may be of limited usefulness in the future.

Considering use of these sampling methods in the resource-limited context of Haiti, purposive sampling tested a higher number of households (50 per index case compared to 20) and therefore was more resource-intensive for both field staff hours and study materials, but random sampling was more time-consuming overall due to the need for a census of the index EA in order to select a random sample, which took 2.2 days per EA on average. Interestingly, purposive testing of only the closest 20 households to the index case yielded a similar percent positivity as the closest 50 households in sub-analyses, which corresponds to previous findings in Haiti that additional LF cases may be clustered close to the index case [22]. Purposive sampling of fewer households may therefore be an option in the future to save resources, although more research is needed to confirm this across settings and species of LF. Random sampling might also not be as time intensive if countries have already performed the appropriate census in advance, if satellite mapping could be used to enumerate households, or if alternatives to random sampling such as segmentation could be used. As a whole, both random and purposive sampling had merits and continue to be potential methods for TAS follow-up.

In this setting, having a higher number of index cases was also correlated with a higher percent positivity. Four of the original index cases were found in Miragoane, and percent positivity in this geographic zone was 2.4% compared to 0.0–1.8% in the other 4 zones where only one index case was found. Despite this result, sample sizes were too small to draw definitive conclusions, and percent positivity approached the 2% cutoff in the urban zone of Anse-a-Veau despite having only one index case. These observations indicate that, although having multiple index cases in an area might suggest heightened chance of ongoing transmission, at this time the conservative choice still would be to follow up every positive case found in TAS.

Of importance for the LF elimination effort in Haiti, the CFA prevalence of 2.4% in Miragoane was higher than the 2% cutoff typically needed in pre-TAS sentinel checks to initiate the TAS process and stop MDA in regions with *Culex* vectors [4]. The two CFA-positive children under 7 years were also found in Miragoane, which is concerning since MDA was stopped in Nippes eight years prior and might be a further indication of ongoing transmission. This is a change from historical data, as Miragoane had no positive LF cases during initial mapping; however, the dramatic urbanization of the commune and influx of people from rural areas might have resulted in a higher concentration of cases and thereby allowed low levels of transmission despite the pressure of MDA. As shown in previous research, urban areas can have unique barriers to LF elimination, including high community mobility and lower compliance with MDA [23,24]. As a result of this study’s findings, the Haiti National Program to Eliminate LF has decided to adjust the practice of combining all the Nippes Department communes for the upcoming TAS 3 and will evaluate Miragoane separately from the rest of the department. This will likely provide a more sensitive result and will identify whether the commune needs to resume MDA.

This study was also enriched by antibody testing, which provide a more thorough picture of LF in Nippes. As seen in other studies [25,26], a higher proportion of participants were seropositive than CFA-positive since antibody responses may be seen in both past and present LF infection [27]. Seropositivity was not significantly different by geographic zone and was not correlated with number of index cases, but was higher among males, older adults, and among participants who reported not sleeping under a bed net. This might indicate higher prevalence of lifetime infection in these groups, consistent with other studies [28]. Of note, seropositivity in this population was approximately 3% in both children under 10 years and children aged 10–19 years. Seropositivity in the youngest age group could indicate persistent or recurrent LF transmission since infection was likely acquired after MDA was stopped eight years prior to the study and it takes approximately three years to develop antibodies after initial infection [29]. Antibody response to Wb123 has also been correlated with molecular xenomonitoring results in prior studies, further suggesting that ongoing transmission may be occurring [25]. However, this could also indicate residual seropositivity following interruption of transmission in the older children. Continuing vigilance in this population is warranted to determine if antibody responses are indicative of ongoing transmission.

Similar to previous investigations [30–32], individual-level concordance of Wb123-specific antibody and CFA antigen was poor in this investigation. Eighty-one participants (4%) were antibody positive only, which can be explained by past infection and persistent antibody response. However, 23 participants (1%) were CFA-positive only, which constituted a high proportion of the total 29 CFA-positives. This could potentially be due to recent infection or a less durable antibody response in some individuals [27,29], but further research is needed to characterize the relationship between LF antibody and antigen response to better understand the utility of these markers during post-MDA surveillance. Therefore, antibody results appear to primarily be useful at the population level to describe patterns and trends in transmission but may not be appropriate for individual diagnosis, while antigen detection remains the gold standard for both diagnosis and surveillance.

This study had several limitations. Individuals in sampled households could have been missed if they were away during household visits, and responses to the survey could have been inaccurate if respondents did not sufficiently recall their history or provided answers they thought interviewers preferred. Due to the small sample size of positive participants and high number of zero cells, there was insufficient power to conduct multivariate analyses, including more complex analyses comparing sampling methods or antigen vs. antibody results. Furthermore, due to varying household density in urban vs. rural zones, the distance of the 50 closest household to the index case varied greatly during purposive sampling. In urban areas, the diameter of the purposive circle was as small as 135 m, but it ranged up to 2 km in rural areas, so an ideal minimum distance for sampling could not be established. This issue was also seen to a lesser extent in random sampling, since the size of the census EA also varied by household density and was as small as 0.02 square kilometers in urban settings. Future studies could sample all households within a set diameter in order to determine a minimum sampling distance.

Another limitation of this study is the possibility that the cutoff values for the ELISA OD values were inaccurate. Due to the absence of well-characterized panels for neglected tropical diseases, it is often challenging to determine cutoffs for serological assays. The method used in this study represents fitting two distributions to the OD data with the assumption that there is limited overlap between the results for true positive and negative samples. Although this method is becoming standard in neglected tropical disease research, it is unknown to what extent the assumptions are met, particularly given the unknown duration of antibody responses to these pathogens. Future studies and meta-analyses can better confirm the appropriateness of this method.

Despite these limitations, this study provides some of the first systematic analyses on follow-up of LF CFA-positive children identified by TAS. Two sampling methods were demonstrated to be achievable in resource-limited settings, and both identified strong signs of ongoing transmission in the large urban commune of Miragoane. This area of potential transmission after the cessation of MDA has the potential to disrupt the local program to eliminate LF in Haiti and further demonstrates the importance of TAS follow-up for the Global Program to Eliminate Lymphatic Filariasis. While this study will help inform standardized guidelines for post-TAS surveillance, more research is needed, and additional results including cost-efficacy analyses from forthcoming post-TAS surveillance analyses from the Philippines, Burkina Faso, and Nepal will continue to explore best methods for TAS follow up.

## Supporting information

S1 Text Detailed methods for ELISA antibody testing. (DOCX)

S1 Table Number and percent of participants who tested positive for circulating filarial antigen by index case and sampling method, Nippes Department, Haiti, July-August 2019. Purposive sampling N = 1,221; random sampling index case enumeration area (EA) N = 381; random sampling neighbor EA N = 500.
(DOCX)

S2 Table Number and percent of participants who tested serology positive by index case and sampling method, Nippes Department, Haiti, July-August 2019. N = 1,914. (DOCX)

S1 Fig Circulating filarial antigen (CFA) prevalence by geographic zone, index case, and number of purposively sampled households by increasing distance in meters from index case household in comparison to households random sampled in the index case enumeration area (EA) where available, Nippes Department, Haiti, July-August 2019. (TIF)

S2 Fig Circulating filarial antigen (CFA) prevalence by geographic zone and index case by increasing 10-house band of increasing distance from index case household, Nippes Department, Haiti, July-August 2019. Each band comprised only the 10 households in that distance band (e.g. 1–10, 11–20, 21–30, etc.)
(TIF)

S3 Fig Log normalized mean optical density (OD) values for IgG4 antibodies against the recombinant Wb123 antigen by participant characteristics and circulating filarial antigen (CFA) results. Navy color indicates CFA-negative participants, and pink color indicates CFA-positive participants. Red line indicates cutoff for seropositivity.
(TIF)
