# Supplementary material for: Positive-case follow up for lymphatic filariasis after a transmission assessment survey in Haiti
Source: PLoS Negl Trop Dis. 2022 Feb 25;16(2):e0010231. doi: 10.1371/journal.pntd.0010231 (PMC8906642; doi:10.1371/journal.pntd.0010231)
Supplement: S1 Text — (DOCX) [file pntd.0010231.s001.docx]

Supporting Information S1 Text

Detailed methods for ELISA antibody testing

The Filaria Detect^TM^ IgG4 ELISA kit (InBios, Seattle, WA), a direct enzyme immunoassay, was used to detect IgG4 antibodies against the recombinant Wb123 antigen in dried blood spot (DBS) samples. The assay was performed according to the standard operating procedure provided by the manufacturer with minor modifications. Briefly, one blood spot extension (10 µL whole blood) was eluted in 250 µL of kit provided sample buffer to yield a 1:50 final serum dilution then stored overnight at 4 °C. In addition, five controls were prepared, also diluted in sample buffer: a kit-provided high positive, low positive and negative control (each at 1:75 dilution), and two external positive controls available at CDC, used to standardize results across plates (H3 at 1:1500 dilution and H19 at 1:900 dilution; not provided in the kit). The next day, samples and controls were tested in duplicate by adding 100 µL of eluate to each plate well. Plates were sealed and incubated at 37 °C for 30 minutes, then washed 6 times using an automated plate washer and kit-provided wash buffer. Mouse anti-human IgG4 conjugated with horseradish peroxidase was added to each well at a 1:100 dilution, then incubated again sealed at 37 °C for 30 minutes. After another wash cycle, 100 µL of tetramethylbenzidine (TMB) was added to each well and plates developed at room temperature in the dark for 13 minutes (as determined by a TMB optimization procedure conducted at the beginning of the study unique to the specific lot of kits used). 50 µL of kit-provided stop solution was added to each well to stop the reaction; plates incubated one minute uncovered then were read on a microplate reader at 450 nm. To compare optical density (OD) values across plates the OD values were normalized by dividing the mean OD of the sample by the mean OD of the H3-positive control from the same plate.
