# Supplementary material for: Positive-case follow up for lymphatic filariasis after a transmission assessment survey in Haiti
Source: PLoS Negl Trop Dis. 2022 Feb 25;16(2):e0010231. doi: 10.1371/journal.pntd.0010231 (PMC8906642; doi:10.1371/journal.pntd.0010231)
Supplement: S1 Table — Purposive sampling N = 1,221; random sampling index case enumeration area (EA) N = 381; random sampling neighbor EA N = 500. (DOCX) [file pntd.0010231.s002.docx]

Table S1: Number and percent of participants who tested positive for circulating filarial antigen by index case and sampling method, Nippes Department, Haiti, July-August 2019. Purposive sampling N=1,221; random sampling index case enumeration area (EA) N=381; random sampling neighbor EA N=500.

| **Index Case by Zone** | **Purposive sampling**  **n (%)** | **Random (index)**  **n (%)** | **Random (neighbor) n (%)** | **Total**  **n (%)** |
| --- | --- | --- | --- | --- |
| Miragoane |  |  |  |  |
| 1 (a&b) | 6 (2.2%) | 0 (0.0%) | 1 (1.4%) | 7 (2.0%) |
| 2 | 3 (2.4%) | 5 (9.6%) | 0 (0.0%) | 8 (3.4%) |
| 3 | 4 (2.7%) | 1 (2.6%) | 1 (0.9%) | 6 (2.3%) |
| L’Asile |  |  |  |  |
| 4 | 0 (0.0%) | 1 (2.0%) | 0 (0.0%) | 1 (0.4%) |
| Plaisance du Sud |  |  |  |  |
| 5 | 0 (0.0%) | 0 (0.0%) | 0 (0.0%) | 0 (0.0%) |
| Petit-Trou de Nippes |  |  |  |  |
| 6 | 0 (0.0%) | 0 (0.0%) | 0 (0.0%) | 0 (0.0%) |
| Anse-a-Veau rural |  |  |  |  |
| 7 | 2 (1.3%) | 0 (0.0%) | 1 (1.2%) | 3 (1.1%) |
| Anse-a-Veau urban |  |  |  |  |
| 8 | 2 (1.8%) | 2 (3.5%) | 0 (0.0%) | 4 (1.8%) |
| **Total** | **17 (1.4%)** | **9 (2.4%)** | **3 (0.6%)** | **29 (1.5%)** |
