# Supplementary material for: Positive-case follow up for lymphatic filariasis after a transmission assessment survey in Haiti
Source: PLoS Negl Trop Dis. 2022 Feb 25;16(2):e0010231. doi: 10.1371/journal.pntd.0010231 (PMC8906642; doi:10.1371/journal.pntd.0010231)
Supplement: S2 Table — (DOCX) [file pntd.0010231.s003.docx]

Table S2: Number and percent of participants who tested serology positive by index case and sampling method, Nippes Department, Haiti, July-August 2019. N=1,914.

| **Index Case** | **Purposive sampling**  **n (%)** | **Random (index)**  **n (%)** | **Random (neighbor) n (%)** | **Total***  **n (%)** |
| --- | --- | --- | --- | --- |
| Miragoane |  |  |  |  |
| 1 (a&b) | 19 (7.1%) | 3 (5.7%) | 0 (0.0%) | 19 (5.4%) |
| 2 | 7 (5.6%) | 3 (6.0%) | 0 (0.0%) | 10 (4.2%) |
| 3 | 11 (7.5%) | 2 (5.3%) | 3 (2.8%) | 14 (5.3%) |
| L’Asile |  |  |  |  |
| 4 | 6 (3.9%) | 0 (0.0%) | 3 (3.5%) | 9 (3.4%) |
| Plaisance du Sud |  |  |  |  |
| 5 | 5 (3.6%) | 2 (5.1%) | 0 (0.0%) | 6 (3.8%) |
| Petit-Trou de Nippes |  |  |  |  |
| 6 | 6 (5.3%) | 3 (8.1%) | 0 (0.0%) | 9 (6.0%) |
| Anse-a-Veau rural |  |  |  |  |
| 7 | 5 (3.2%) | 1 (2.0%) | 8 (9.2%) | 13 (4.9%) |
| Anse-a-Veau urban |  |  |  |  |
| 8 | 3 (2.7%) | 1 (1.7%) | 2 (2.8%) | 6 (2.7%) |
| **Total** | **62 (5.1%)** | **15 (4.0%)** | **16 (3.2%)** | **86 (4.5%)** |

*Columns may add to more than total if individuals were sampled by both purposive and random sampling
