# Supplementary figures and images for: Positive-case follow up for lymphatic filariasis after a transmission assessment survey in Haiti
Source: PLoS Negl Trop Dis. 2022 Feb 25;16(2):e0010231. doi: 10.1371/journal.pntd.0010231 (PMC8906642; doi:10.1371/journal.pntd.0010231)

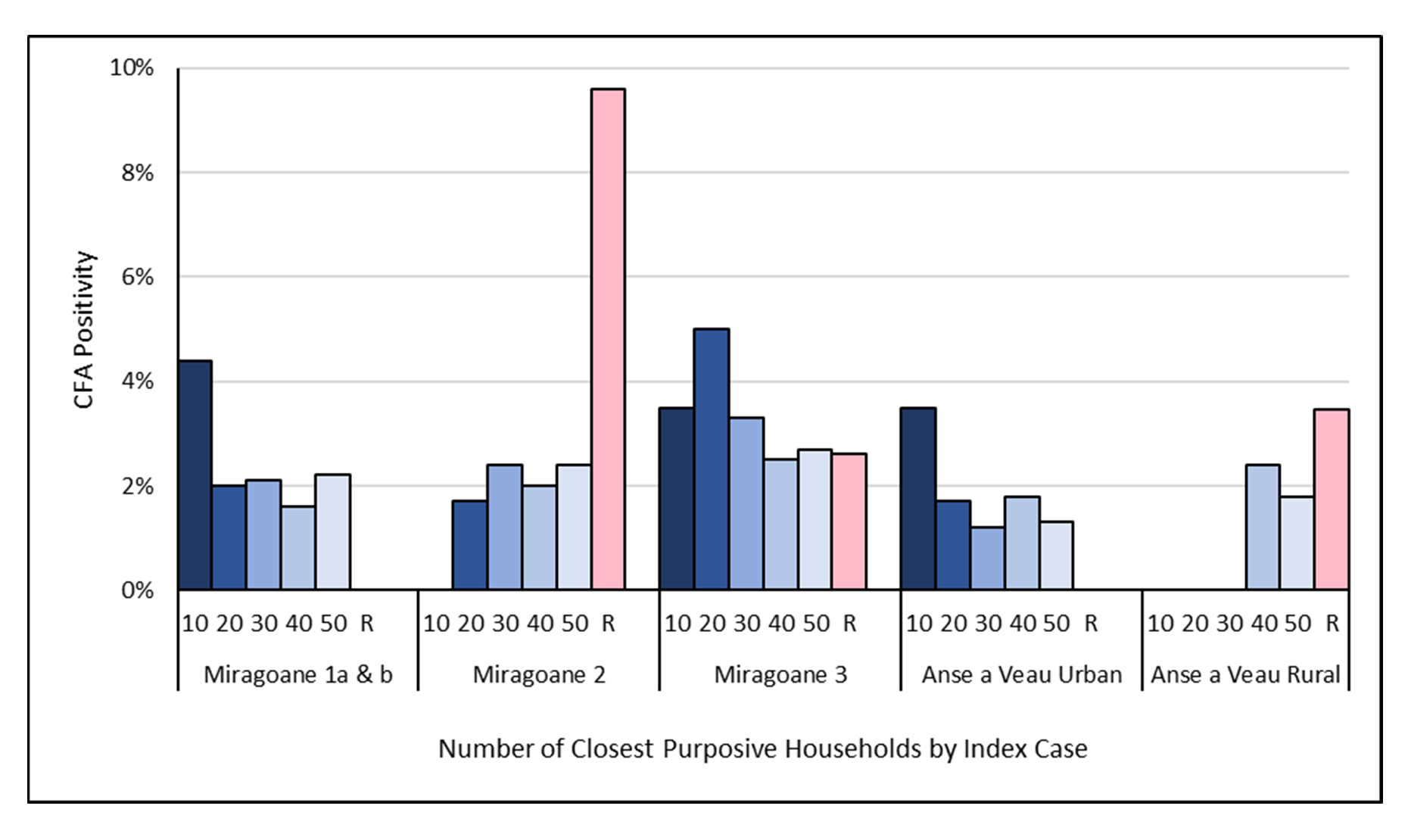

Supplement: S1 Fig — (TIF) [file pntd.0010231.s004.tif]

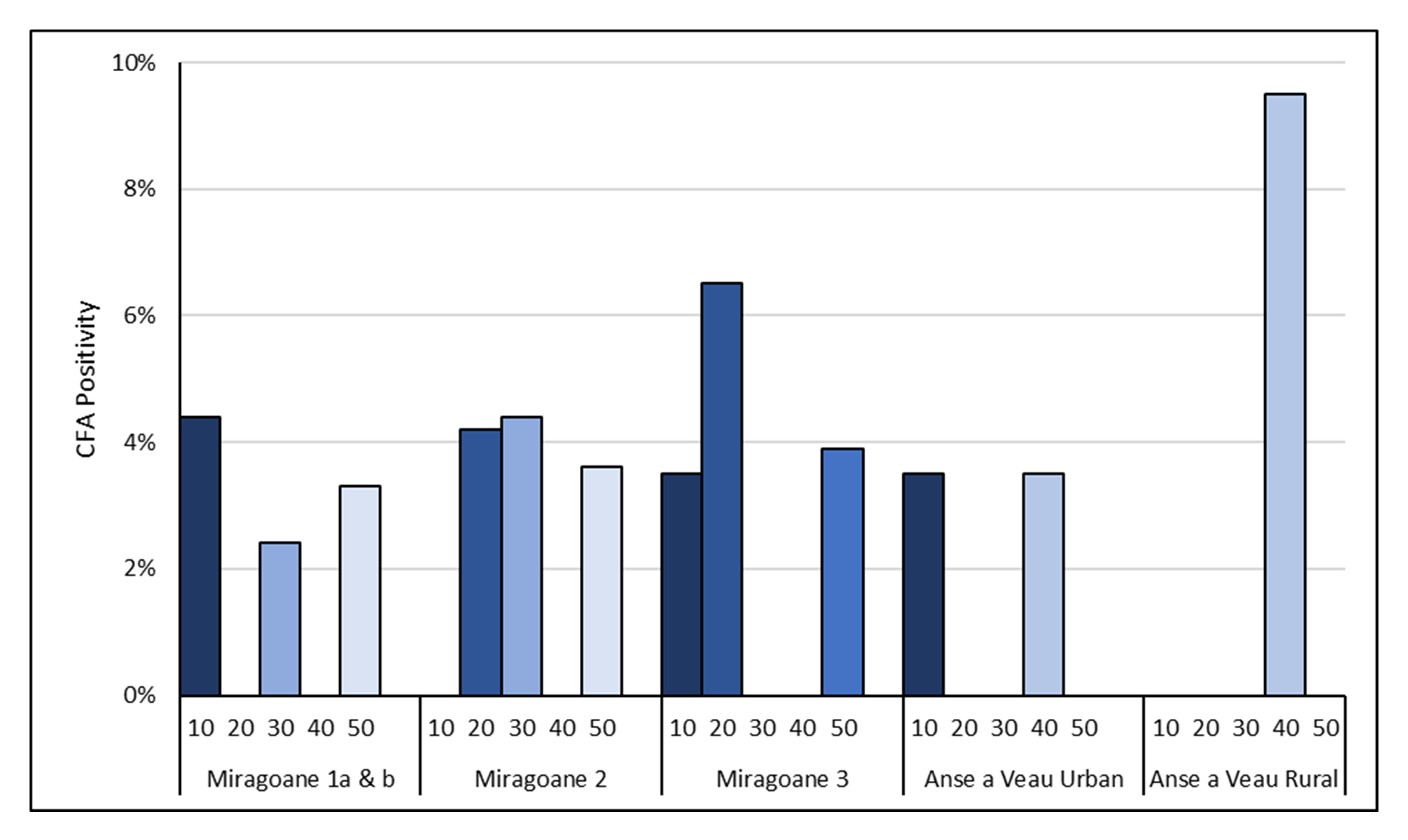

Supplement: S2 Fig — Each band comprised only the 10 households in that distance band (e.g. 1–10, 11–20, 21–30, etc.) (TIF) [file pntd.0010231.s005.tif]

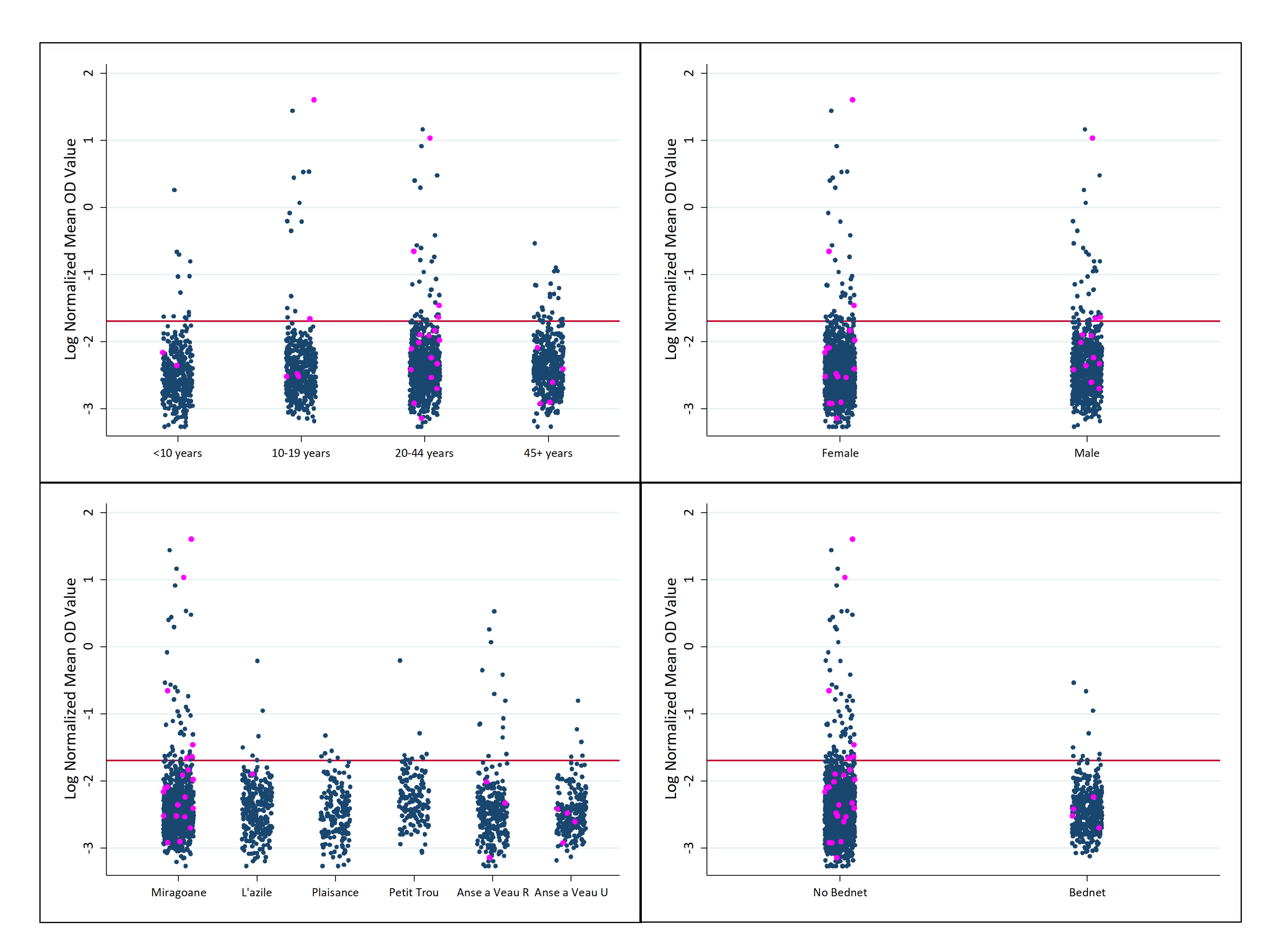

Supplement: S3 Fig — Navy color indicates CFA-negative participants, and pink color indicates CFA-positive participants. Red line indicates cutoff for seropositivity. (TIF) [file pntd.0010231.s006.tif]
